# Dapagliflozin and linagliptin combination therapy improves time in range and reverses hepatic steatosis in patients with type 2 diabetes and hypertension

**DOI:** 10.3389/fendo.2026.1802641

**Published:** 2026-05-11

**Authors:** WenXia Li, RuRan Li, ShanShan Li, Zhen Li, JingSu Ma, Fei Wu, ChaoQin Chen

**Affiliations:** Department of Endocrinology and Metabolism, Hainan Medical University Affiliated Danzhou People’s Hospital, Danzhou, Hainan, China

**Keywords:** dapagliflozin, hypertension, linagliptin, time in range, type 2 diabetes

## Abstract

**Background:**

Type 2 diabetes mellitus (T2DM) with comorbid hypertension increases risks of vascular complications, necessitating optimized therapies. This study evaluated whether dapagliflozin combined with linagliptin improves time in range (TIR) and hepatorenal function compared to monotherapy in this population.

**Methods:**

In this prospective observational cohort study at Danzhou People’s Hospital (June 2021–September 2024), 136 patients aged 40–70 years with T2DM (HbA1c 7.5–11.0%) and hypertension were allocated to four groups (n=34 each): standard care (control), dapagliflozin (10 mg/day), linagliptin (5 mg/day), or combination therapy. Outcomes after 12 weeks included TIR via continuous glucose monitoring, blood pressure, lipids, hepatic (ALT, AST, liver-to-spleen ratio via CT), and renal parameters (UACR, eGFR). Data were analyzed using ANOVA, correlations, and regression.

**Results:**

Combination therapy achieved superior TIR (94.86 ± 3.65% vs. ~80% in monotherapies and 81.44% in control; P<0.001), with greater reductions in HbA1c (to 5.52%), fasting glucose, blood pressure (systolic to 124 mmHg), lipids (triglycerides to 0.72 mmol/L), liver enzymes (ALT to 9.74 U/L), and UACR (to 7.36 mg/g), plus higher eGFR (to 122.41 mL/min/1.73m²; all P<0.05 vs. others). TIR correlated with improved hepatorenal markers; combination therapy predicted greatest TIR change (β=7.896, P<0.001).

**Conclusion:**

Dapagliflozin-linagliptin combination offers superior glycemic stability and organ protection, supporting its use for managing T2DM with hypertension.

## Introduction

Type 2 diabetes mellitus (T2DM) prevalence continues to rise globally, with China reporting an adult diabetes prevalence of 12.8% and nearly 200 million affected individuals (1). Hypertension frequently coexists with T2DM, affecting approximately 80% of diabetic patients, while T2DM prevalence in hypertensive populations is 2.5-fold higher than in normotensive individuals (2, 3). This comorbidity accelerates vascular complications and increases healthcare burden, highlighting the need for optimized management strategies in this high-risk population.

Glycemic monitoring has evolved beyond traditional metrics to embrace continuous glucose monitoring (CGM) parameters. Time in Range (TIR), defined as the percentage of time glucose values remain within 70–180 mg/dL, has emerged as a valuable indicator of glycemic control quality (4, 5). A meta-analysis of 18 randomized controlled trials involving 2,577 patients demonstrated that each 10% decrease in TIR corresponded to a 0.8% increase in HbA1c (6). Furthermore, TIR demonstrates predictive value for diabetic complications independent of HbA1c levels (7), supporting its adoption as both a clinical endpoint and risk stratification tool.

Current therapeutic approaches for T2DM with hypertension include sodium-glucose cotransporter-2 (SGLT-2) inhibitors and dipeptidyl peptidase-4 (DPP-4) inhibitors, which offer complementary mechanisms of action. Dapagliflozin, an SGLT-2 inhibitor, reduces blood glucose through insulin-independent renal glucose excretion while providing cardiovascular and renal benefits. Studies have demonstrated that dapagliflozin reduces urinary albumin excretion and serum creatinine in patients receiving renin-angiotensin system blockade (8), with sustained proteinuria reduction even in those with moderate renal impairment (9). Linagliptin, a DPP-4 inhibitor, enhances glucose-dependent insulin secretion through GLP-1 preservation, offering the advantage of unchanged pharmacokinetics regardless of hepatic or renal function (10).

Despite theoretical synergy between these drug classes, clinical evidence regarding combination therapy effects on glycemic variability and organ protection in T2DM with hypertension remains limited. Most existing research has focused on monotherapy outcomes, leaving a knowledge gap regarding combined treatment benefits on TIR and hepatorenal function. This study aimed to evaluate whether dapagliflozin combined with linagliptin provides superior glycemic control and organ protection compared to monotherapy in patients with T2DM and hypertension, potentially informing treatment optimization for this challenging population.

## Materials and methods

### Study design and setting

This prospective observational cohort study was conducted at the Department of Endocrinology and Metabolism, Danzhou People’s Hospital Affiliated to Hainan Medical University, from June 2021 to September 2024. The study protocol adhered to STROBE (Strengthening the Reporting of Observational Studies in Epidemiology) guidelines and was approved by the Ethics Committee of Danzhou People’s Hospital. All participants provided written informed consent prior to enrollment, and the study was conducted in accordance with the Declaration of Helsinki.

### Study population and recruitment

Consecutive patients diagnosed with T2DM and hypertension were systematically screened during routine clinical visits. Inclusion criteria comprised: (1) age 40–70 years; (2) confirmed diagnosis of T2DM according to American Diabetes Association criteria (11) and hypertension per ESC guidelines (12); (3) suboptimal glycemic control defined as HbA1c between 7.5% and 11.0% despite at least three months of stable glucose-lowering therapy; (4) inadequate blood pressure control (130 mmHg ≤ systolic blood pressure < 160 mmHg and/or 80 mmHg ≤ diastolic blood pressure < 100 mmHg) despite stable antihypertensive therapy for at least 4 weeks.

Exclusion criteria encompassed: (1) other diabetes types or secondary hypertension; (2) documented hypersensitivity to SGLT-2 inhibitors or DPP-4 inhibitors; (3) hematologic conditions affecting HbA1c measurement reliability including hemoglobinopathies, recent blood transfusion within 3 months, or chronic hemolytic anemia; (4) recent exposure to medications with known hepatorenal toxicity within the preceding 8 weeks; (5) significant hepatic impairment (ALT or AST > 2.5 times upper limit of normal) or severe renal impairment (eGFR < 45 mL/min/1.73m²); (6) lifestyle factors compromising study compliance including shift work involving >2 night shifts weekly, planned relocation, or inability to attend follow-up visits.

### Sample size determination

Sample size calculation was performed *a priori* using G*Power software version 3.1, based on detecting between-group differences in the primary outcome, Time in Range (TIR). Previous literature suggested a clinically meaningful TIR difference of 10% with an expected standard deviation of 15%. With an effect size f = 0.25, α = 0.05, statistical power (1-β) = 0.80, and four comparison groups, the minimum required sample size was 128 participants. Accounting for an anticipated 6% attrition rate based on institutional historical data, the final enrollment target was set at 136 participants (34 per group).

### Treatment allocation and intervention protocol

Following baseline assessment, participants were allocated to one of four treatment groups based on physician clinical judgment and patient characteristics, considering contraindications and previous treatment responses. The four groups consisted of: (A) Standard care control group receiving optimized background therapy alone; (B) Dapagliflozin group receiving 10 mg once daily in the morning; (C) Linagliptin group receiving 5 mg once daily; (D) Combination group receiving dapagliflozin 10 mg plus linagliptin 5 mg daily. Medication adherence was monitored through pill counts at 4-week intervals and patient diaries. All participants continued their established background diabetes and hypertension medications throughout the 12-week observation period. Dose adjustments of background medications were permitted only for safety concerns and were documented.

### Clinical and laboratory assessments

#### Baseline characterization

Comprehensive baseline assessment included standardized physical examination, thoracic computed tomography, 12-lead electrocardiography, and complete laboratory evaluation performed within 2 hours of admission. Demographic data, disease duration, cardiovascular risk factors, and comorbidities were systematically recorded using structured case report forms.

#### Blood pressure measurement

Standardized blood pressure measurements followed international guidelines. Measurements were obtained from the right upper arm using a calibrated mercury sphygmomanometer after 5 minutes of seated rest. Three consecutive readings were taken at 2-minute intervals, with the mean value calculated for analysis. Measurements were performed by trained personnel blinded to treatment allocation, consistently between 8:00 and 10:00 AM under fasting conditions.

#### Continuous glucose monitoring

Glycemic variability assessment utilized a silicon-based continuous glucose monitoring system (CGM System, Shenzhen, China). Sensor calibration was performed according to manufacturer specifications with capillary glucose measurements twice daily. Pre-treatment monitoring lasted 72 hours to establish baseline glycemic patterns, while post-treatment assessment extended to 14 days to capture comprehensive glycemic profiles. Data completeness required >70% sensor readings, with missing data managed through validated interpolation methods.

#### Laboratory procedures

Venous blood samples were collected after 12-hour overnight fasting at baseline and week 12. Samples were processed within 2 hours of collection to minimize pre-analytical variability. Following centrifugation at 3600 rpm for 10 minutes at 4 °C, serum aliquots were stored at -80 °C until batch analysis. Biochemical parameters including FBG, HbA1c, lipid profile (TC, TG, LDL-C, HDL-C), hepatic enzymes (ALT, AST), and serum creatinine were measured using an AU600 automated analyzer (Beckman-Olympus, USA) with commercial reagent kits (Pars Azmoon, Iran). Inter-assay coefficients of variation were <3% for all parameters. Twenty-four-hour urine collections were performed for albumin-to-creatinine ratio determination, with eGFR calculated using the MDRD equation (13). Urine collection adequacy was verified through creatinine excretion rates.

#### Hepatic imaging assessment

Non-contrast abdominal CT imaging was performed using a GE 64-slice spiral scanner (GE Medical Systems, Tokyo, Japan) following standardized protocols. Image acquisition parameters included 120 kVp, automated tube current modulation, and 5-mm slice thickness reconstruction. Hepatic steatosis quantification employed the liver-to-spleen attenuation ratio (L/S ratio), with measurements obtained from two 100 mm² regions of interest in the right hepatic lobe, avoiding visible vasculature. All measurements were performed by a single radiologist blinded to clinical data, with intra-observer variability <5%. Hepatic steatosis was defined as L/S ratio <1.0 (14), with ratio improvements indicating reduced hepatic fat content.

### Statistical analysis

Statistical analyses were performed using SPSS version 26.0 (IBM Corp, Chicago, IL, USA). Data distribution normality was assessed using Shapiro-Wilk tests and Q-Q plots. Continuous variables with normal distribution are presented as mean ± standard deviation, analyzed using one-way ANOVA with *post-hoc* LSD tests for pairwise comparisons. Non-normally distributed data are expressed as median (interquartile range) and analyzed using Kruskal-Wallis tests. Categorical variables were compared using chi-square tests or Fisher’s exact tests when expected frequencies were <5.

Spearman correlation analysis evaluated relationships between TIR and metabolic parameters, with correlation strength interpreted as: |r| = 0.8-1.0 (very strong), 0.6-0.8 (strong), 0.4-0.6 (moderate), 0.2-0.4 (weak), 0-0.2 (negligible). Multiple linear regression using stepwise selection (entry P<0.05, removal P>0.10) identified independent predictors of TIR change (ΔTIR). Model assumptions including linearity, homoscedasticity, and absence of multicollinearity (VIF <10) were verified. All tests were two-sided with significance set at P<0.05.

## Results

### Study population and baseline characteristics

**Figure 1:**
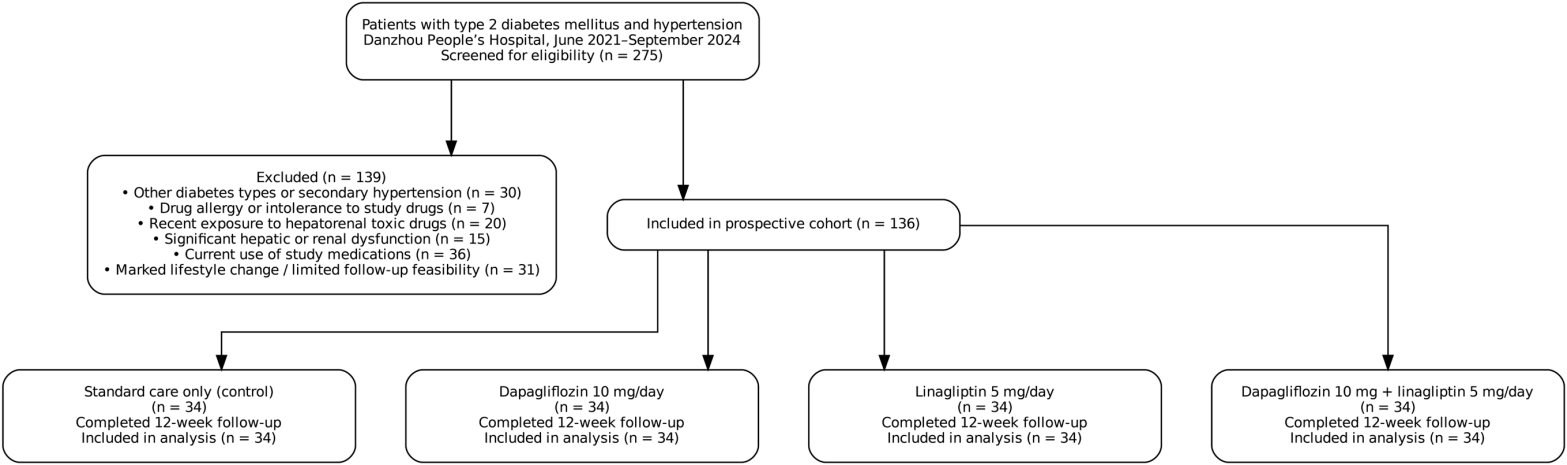
Study flow diagram. Flow chart depicting patient screening, enrollment, and allocation process. Of 275 eligible patients with type 2 diabetes mellitus and hypertension screened between June 2021 and September 2024, 139 were excluded based on predefined criteria: other diabetes types or secondary hypertension (n=30), known allergies to study medications (n=7), recent hepatorenal toxic drug exposure (n=20), significant hepatic or renal dysfunction (n=15), current use of study medications (n=36), and lifestyle factors affecting compliance (n=31). The final cohort of 136 participants was equally allocated to four treatment groups (n=34 each): control (standard care), dapagliflozin monotherapy (10 mg daily), linagliptin monotherapy (5 mg daily), and combination therapy (dapagliflozin 10 mg plus linagliptin 5 mg daily). All participants completed the 12-week treatment period with no dropouts.

Of 275 eligible patients screened during the study period, 139 were excluded based on predefined criteria. The primary reasons for exclusion included alternative diabetes diagnoses or secondary hypertension (n=30), documented medication allergies (n=7), recent hepatorenal toxic drug exposure (n=20), significant organ dysfunction (n=15), current use of study medications (n=36), and lifestyle factors affecting study compliance (n=31). The final cohort comprised 136 participants equally distributed across four treatment groups (n=34 per group), with complete follow-up achieved in all participants (**Figure 1**).

Baseline demographic and clinical characteristics demonstrated homogeneity across treatment groups (**Table 1**). The overall cohort had a mean age of 61.0 ± 6.2 years with balanced gender distribution (50% male). Participants presented with established disease, averaging 12.5 ± 4.1 years since T2DM diagnosis and 10.4 ± 3.7 years of hypertension. Cardiovascular risk factors were prevalent, including smoking in 16.9% and alcohol consumption in 19.9% of participants. Family history revealed diabetes in 54.4% and hypertension in 44.9% of cases. Comorbidities reflected typical diabetic complications, with diabetic retinopathy in 22.8%, peripheral neuropathy in 19.1%, and coronary heart disease in 30.1% of participants. Mean BMI of 26.0 ± 2.7 kg/m² indicated overweight status across all groups.

**Table 1:** Baseline demographic and clinical characteristics of study participants.

Characteristic	Control group (n=34)	Dapagliflozin group (n=34)	Linagliptin group (n=34)	Combination group (n=34)	F/χ²	P value
Demographics						
Age, years	61.2 ± 5.4	60.9 ± 5.9	61.7 ± 6.5	59.9 ± 7.3	0.510	0.676
Male sex, n (%)	15 (44.1)	18 (52.9)	16 (47.1)	19 (55.9)	1.176	0.758
BMI, kg/m²	26.2 ± 2.8	25.8 ± 2.6	26.3 ± 2.7	26.0 ± 3.0	0.237	0.871
Disease duration						
Type 2 diabetes, years	12.4 ± 4.1	12.1 ± 3.7	13.1 ± 5.0	12.7 ± 3.9	0.328	0.805
Hypertension, years	10.9 ± 3.0	9.9 ± 3.4	11.2 ± 4.0	10.5 ± 4.5	0.776	0.510
Risk factors, n (%)						
Current smoking	4 (11.8)	6 (17.6)	6 (17.6)	7 (20.6)	0.994	0.802
Alcohol consumption	7 (20.6)	5 (14.7)	7 (20.6)	8 (23.5)	0.878	0.831
Family history of diabetes	18 (52.9)	19 (55.9)	17 (50.0)	20 (58.8)	0.593	0.898
Family history of hypertension	15 (44.1)	14 (41.2)	16 (47.1)	16 (47.1)	0.327	0.955
Comorbidities, n (%)						
Diabetic retinopathy	8 (23.5)	7 (20.6)	7 (20.6)	9 (26.5)	0.460	0.928
Diabetic peripheral neuropathy	6 (17.6)	8 (23.5)	5 (14.7)	7 (20.6)	0.951	0.813
Coronary heart disease	9 (26.5)	11 (32.4)	10 (29.4)	11 (32.4)	0.384	0.944
Atherosclerosis	14 (41.2)	15 (44.1)	13 (38.2)	16 (47.1)	0.601	0.896
Baseline laboratory values						
FBG, mmol/L	10.3 ± 1.9	9.9 ± 1.4	10.0 ± 2.1	10.7 ± 2.0	1.256	0.292
SBP, mmHg	151.4 ± 8.5	154.2 ± 9.1	150.5 ± 6.5	153.5 ± 8.3	1.541	0.207
DBP, mmHg	96.4 ± 4.6	97.7 ± 5.1	96.6 ± 5.4	95.7 ± 5.4	0.969	0.409
Lipid profile, mmol/L						
Total cholesterol	5.58 ± 1.54	5.47 ± 1.60	5.61 ± 1.57	5.50 ± 1.46	0.060	0.981
Triglycerides	2.62 ± 0.52	2.58 ± 0.46	2.55 ± 0.51	2.59 ± 0.59	0.117	0.950
LDL-cholesterol	3.08 ± 0.63	3.12 ± 0.60	3.17 ± 0.55	3.14 ± 0.58	0.140	0.936
HDL-cholesterol	1.26 ± 0.21	1.23 ± 0.19	1.26 ± 0.24	1.28 ± 0.21	0.378	0.769

Data are presented as mean ± SD or n (%). P values derived from one-way ANOVA for continuous variables and χ² test for categorical variables. Abbreviations: BMI, body mass index; DBP, diastolic blood pressure; FBG, fasting blood glucose; HDL, high-density lipoprotein; LDL, low-density lipoprotein; SBP, systolic blood pressure.

### Glycemic control and time in range outcomes

**Figure 2:**
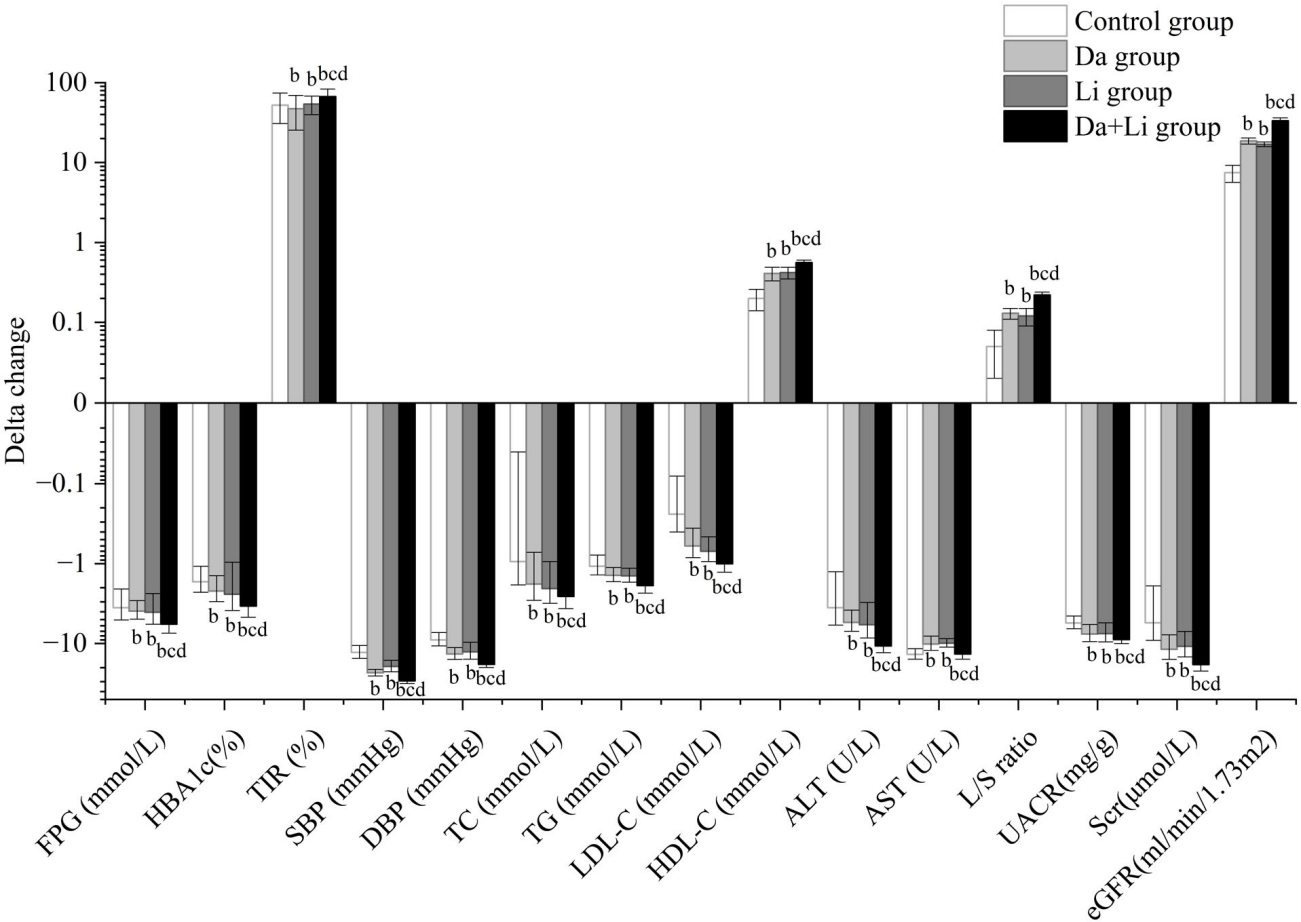
Changes in metabolic parameters from baseline to week 12 across treatment groups. A bar graph illustrating the magnitude of change (week 12 minus baseline) in key metabolic parameters for each treatment group. Positive values indicate parameter increases; negative values indicate decreases. The plotted parameters span glycemic control (FPG, HbA1c, TIR), blood pressure (SBP, DBP), lipid profiles (TC, TG, LDL-C, HDL-C), hepatic function markers (ALT, AST, L/S ratio), and renal function indicators (UACR, Scr, eGFR). Error bars represent standard error of the mean. Statistical comparisons: ^b^P<0.05 versus control group; ^c^P<0.05 versus dapagliflozin group; ^d^P<0.05 versus linagliptin group. The combination therapy group demonstrated significantly greater improvements across all parameter categories compared to monotherapy and control groups. Abbreviations: ALT, alanine aminotransferase; AST, aspartate aminotransferase; DBP, diastolic blood pressure; eGFR, estimated glomerular filtration rate; FPG, fasting plasma glucose; HbA1c, glycated hemoglobin A1c; HDL-C, high-density lipoprotein cholesterol; LDL-C, low-density lipoprotein cholesterol; L/S ratio, liver-to-spleen attenuation ratio; SBP, systolic blood pressure; Scr, serum creatinine; TC, total cholesterol; TG, triglycerides; TIR, time in range; UACR, urinary albumin-to-creatinine ratio.

The primary endpoint of TIR improvement demonstrated marked treatment-dependent variations after 12 weeks (**Table 2**, **Figure 2**). Baseline TIR values were comparably suboptimal across groups, ranging from 27.74% to 31.89%. Following intervention, all treatment groups achieved substantial TIR improvements, with the combination therapy demonstrating superior efficacy. The control group achieved a TIR of 81.44 ± 10.32%, while monotherapy groups reached comparable levels (dapagliflozin: 79.22 ± 9.27%; linagliptin: 81.72 ± 5.51%). The combination therapy group attained significantly higher TIR at 94.86 ± 3.65%, representing the greatest absolute improvement from baseline (P<0.001 versus all comparators).

**Table 2:** Changes in metabolic parameters, hepatic function, and renal function after 12-week treatment,.

Parameter	Time point	Control group (n=34)	Dapagliflozin group (n=34)	Linagliptin group (n=34)	Combination group (n=34)
Glycemic control					
FBG, mmol/L	Baseline	10.31 ± 1.90	9.94 ± 1.37	10.00 ± 2.09	10.72 ± 1.97
	Week 12	6.74 ± 0.40*	6.02 ± 0.35*†	5.96 ± 0.42*†	5.21 ± 0.29*†‡§
HbA1c, %	Baseline	8.83 ± 1.22	8.75 ± 1.29	9.02 ± 1.91	8.92 ± 1.63
	Week 12	7.15 ± 0.62*	6.54 ± 0.51*†	6.61 ± 0.45*†	5.52 ± 0.36*†‡§
TIR, %	Baseline	28.91 ± 18.10	31.89 ± 18.89	27.74 ± 13.57	29.92 ± 16.85
	Week 12	81.44 ± 10.32*	79.22 ± 9.27*	81.72 ± 5.51*	94.86 ± 3.65*†‡§
Blood pressure					
SBP, mmHg	Baseline	151.38 ± 8.54	154.24 ± 9.08	150.53 ± 6.46	153.47 ± 8.31
	Week 12	138.47 ± 7.10*	131.03 ± 8.18*†	131.24 ± 8.87*†	124.18 ± 7.01*†‡§
DBP, mmHg	Baseline	96.35 ± 4.62	97.74 ± 5.12	96.59 ± 5.41	95.65 ± 5.35
	Week 12	87.35 ± 3.47*	84.24 ± 3.20*†	83.94 ± 3.89*†	77.53 ± 4.94*†‡§
Lipid profile, mmol/L					
Total cholesterol	Baseline	5.58 ± 1.54	5.47 ± 1.60	5.61 ± 1.57	5.50 ± 1.46
	Week 12	4.65 ± 0.61*	3.66 ± 0.53*†	3.58 ± 0.46*†	2.94 ± 0.39*†‡§
Triglycerides	Baseline	2.62 ± 0.52	2.58 ± 0.46	2.55 ± 0.51	2.59 ± 0.59
	Week 12	1.54 ± 0.23*	1.18 ± 0.18*†	1.13 ± 0.24*†	0.72 ± 0.11*†‡§
LDL-cholesterol	Baseline	3.08 ± 0.63	3.12 ± 0.60	3.17 ± 0.55	3.14 ± 0.58
	Week 12	2.84 ± 0.40*	2.52 ± 0.35*†	2.47 ± 0.31*†	2.15 ± 0.28*†‡§
HDL-cholesterol	Baseline	1.26 ± 0.21	1.23 ± 0.19	1.26 ± 0.24	1.28 ± 0.21
	Week 12	1.46 ± 0.15*	1.64 ± 0.12*†	1.68 ± 0.17*†	1.84 ± 0.22*†‡§
Hepatic function					
ALT, U/L	Baseline	20.28 ± 3.68	19.36 ± 3.22	19.83 ± 4.00	20.41 ± 3.40
	Week 12	16.73 ± 1.40*	13.94 ± 1.62*†	14.02 ± 1.25*†	9.74 ± 1.10*†‡§
AST, U/L	Baseline	25.67 ± 4.19	26.03 ± 4.05	25.75 ± 3.82	25.94 ± 3.64
	Week 12	21.03 ± 2.13*	16.02 ± 2.00*†	15.88 ± 2.60*†	12.46 ± 1.50*†‡§
L/S ratio	Baseline	0.89 ± 0.15	0.86 ± 0.12	0.87 ± 0.14	0.88 ± 0.15
	Week 12	0.94 ± 0.11*	0.99 ± 0.10*†	0.99 ± 0.11*†	1.11 ± 0.13*†‡§
Renal function					
UACR, mg/g	Baseline	17.15 ± 3.60	17.34 ± 3.71	16.64 ± 3.12	16.29 ± 2.94
	Week 12	11.64 ± 2.56*	9.72 ± 1.86*†	9.11 ± 1.13*†	7.36 ± 1.85*†‡§
Serum creatinine, μmol/L	Baseline	78.45 ± 6.17	78.66 ± 7.04	78.03 ± 6.74	78.94 ± 7.54
	Week 12	72.93 ± 2.52*	66.85 ± 3.04*†	67.18 ± 2.90*†	60.63 ± 3.72*†‡§
eGFR, mL/min/1.73m²	Baseline	86.50 ± 13.53	88.98 ± 14.81	87.75 ± 14.57	88.97 ± 14.77
	Week 12	93.96 ± 11.73*	107.69 ± 13.13*†	104.69 ± 13.43*†	122.41 ± 17.70*†‡§

Data are presented as mean ± SD. *P<0.05 vs baseline within group; †P<0.05 vs control group at week 12; ‡P<0.05 vs dapagliflozin group at week 12; §P<0.05 vs linagliptin group at week 12. Abbreviations: ALT, alanine aminotransferase; AST, aspartate aminotransferase; DBP, diastolic blood pressure; eGFR, estimated glomerular filtration rate; FBG, fasting blood glucose; HbA1c, glycated hemoglobin A1c; HDL, high-density lipoprotein; LDL, low-density lipoprotein; L/S ratio, liver-to-spleen attenuation ratio; SBP, systolic blood pressure; TIR, time in range; UACR, urinary albumin-to-creatinine ratio.

Concurrent glycemic parameters corroborated these findings. FBG reductions paralleled TIR improvements, with the combination group achieving the lowest post-treatment value of 5.21 ± 0.29 mmol/L compared to control (6.74 ± 0.40 mmol/L) and monotherapy groups (dapagliflozin: 6.02 ± 0.35 mmol/L; linagliptin: 5.96 ± 0.42 mmol/L; all P<0.05). Similarly, HbA1c decreased from comparable baseline values (8.75-9.02%) to 5.52 ± 0.36% in the combination group, significantly lower than control (7.15 ± 0.62%) and both monotherapies (P<0.001).

### Blood pressure and lipid metabolism

Cardiovascular risk parameters showed comprehensive improvement across all intervention groups, with combination therapy yielding optimal results (**Table 2**). Systolic blood pressure decreased from baseline values exceeding 150 mmHg to 124.18 ± 7.01 mmHg in the combination group, representing significantly greater reduction than control (138.47 ± 7.10 mmHg) or either monotherapy (dapagliflozin: 131.03 ± 8.18 mmHg; linagliptin: 131.24 ± 8.87 mmHg; P<0.05 for all comparisons). Diastolic pressure demonstrated parallel improvements, reaching 77.53 ± 4.94 mmHg with combination therapy versus 87.35 ± 3.47 mmHg in controls (P<0.001).

Lipid profiles underwent favorable remodeling across all treatment groups, with the combination regimen producing the most pronounced effects. Total cholesterol decreased from baseline 5.50 ± 1.46 mmol/L to 2.94 ± 0.39 mmol/L in the combination group, substantially lower than control (4.65 ± 0.61 mmol/L) and monotherapy outcomes (P<0.001). Triglyceride reduction was particularly striking with combination therapy, declining to 0.72 ± 0.11 mmol/L from baseline 2.59 ± 0.59 mmol/L. LDL-C decreased to 2.15 ± 0.28 mmol/L while HDL-C increased to 1.84 ± 0.22 mmol/L in the combination group, both significantly different from other groups (P<0.001).

### Hepatic function and steatosis assessment

Hepatic parameters demonstrated treatment-associated improvements, with combination therapy conferring maximal benefit (**Table 2**; **Figure 2**). ALT levels decreased from baseline 20.41 ± 3.40 U/L to 9.74 ± 1.10 U/L in the combination group, representing greater reduction than control (16.73 ± 1.40 U/L) or monotherapy groups (dapagliflozin: 13.94 ± 1.62 U/L; linagliptin: 14.02 ± 1.25 U/L; P<0.001). AST followed similar patterns, decreasing to 12.46 ± 1.50 U/L with combination therapy versus 21.03 ± 2.13 U/L in controls.

Hepatic steatosis assessment through L/S ratio revealed progressive improvement across treatment intensities. Baseline ratios below 1.0 indicated prevalent hepatic steatosis across all groups. The combination group achieved the highest post-treatment L/S ratio of 1.11 ± 0.13, surpassing the steatosis threshold and significantly exceeding control (0.94 ± 0.11) and monotherapy values (both 0.99 ± 0.10-0.11; P<0.001). These imaging findings corroborated biochemical improvements, suggesting reduced hepatic fat accumulation with combination therapy.

### Renal function parameters

Renal function markers exhibited consistent improvement patterns favoring combination therapy (**Table 2**). Urinary albumin-to-creatinine ratio decreased from baseline 16.29 ± 2.94 mg/g to 7.36 ± 1.85 mg/g in the combination group, representing greater albuminuria reduction than control (11.64 ± 2.56 mg/g) or monotherapy groups (P<0.001). Serum creatinine declined from 78.94 ± 7.54 μmol/L to 60.63 ± 3.72 μmol/L with combination treatment, significantly lower than all comparators.

Estimated GFR improvements reflected enhanced renal function across all treatment groups, with combination therapy achieving the greatest increase. From baseline 88.97 ± 14.77 mL/min/1.73m², the combination group reached 122.41 ± 17.70 mL/min/1.73m² post-treatment, substantially higher than control (93.96 ± 11.73 mL/min/1.73m²) and monotherapy outcomes (dapagliflozin: 107.69 ± 13.13; linagliptin: 104.69 ± 13.43 mL/min/1.73m²; P<0.001).

### Correlations between TIR and metabolic parameters

Post-treatment correlation analysis revealed significant associations between TIR and multiple metabolic indicators (**Table 3**). Glycemic parameters showed expected moderate negative correlations with TIR, including FBG (r = -0.454, P<0.001) and HbA1c (r = -0.466, P<0.001). Among blood pressure measurements, only diastolic pressure demonstrated significant correlation with TIR (r = -0.240, P = 0.005), while systolic pressure showed no significant association (P = 0.114).

**Table 3:** Correlation between time in range and metabolic parameters after 12-week treatment (n=136).

Variable	Correlation coefficient (r)	P value
Glycemic parameters		
FBG, mmol/L	−0.454	<0.001
HbA1c, %	−0.466	<0.001
Blood pressure		
SBP, mmHg	−0.136	0.114
DBP, mmHg	−0.240	0.005
Hepatic function		
ALT, U/L	−0.480	<0.001
AST, U/L	−0.432	<0.001
L/S ratio	0.309	<0.001
Renal function		
UACR, mg/g	−0.310	<0.001
Serum creatinine, μmol/L	−0.373	<0.001
eGFR, mL/min/1.73m²	0.279	0.001

Spearman correlation coefficients presented. Correlation strength interpretation: |r| = 0.8–1.0, very strong; 0.6–0.8, strong; 0.4–0.6, moderate; 0.2–0.4, weak; 0–0.2, negligible. Abbreviations: ALT, alanine aminotransferase; AST, aspartate aminotransferase; DBP, diastolic blood pressure; eGFR, estimated glomerular filtration rate; FBG, fasting blood glucose; HbA1c, glycated hemoglobin A1c; L/S ratio, liver-to-spleen attenuation ratio; SBP, systolic blood pressure; UACR, urinary albumin-to-creatinine ratio.

Hepatic markers exhibited robust correlations with glycemic stability. TIR demonstrated moderate negative correlations with both ALT (r = -0.480, P<0.001) and AST (r = -0.432, P<0.001), while showing positive correlation with L/S ratio (r = 0.309, P<0.001).

Renal parameters similarly correlated with TIR, showing negative associations with UACR (r = -0.310, P<0.001) and serum creatinine (r = -0.373, P<0.001), while demonstrating positive correlation with eGFR (r = 0.279, P = 0.001).

### Predictors of TIR improvement

Multiple linear regression analysis identified independent factors influencing ΔTIR magnitude (**Table 4**). The final model explained 42.8% of ΔTIR variance (adjusted R² = 0.428, F = 16.24, P<0.001), with no evidence of multicollinearity (all VIF values <3). Treatment allocation emerged as the strongest predictor, with combination therapy demonstrating the highest standardized coefficient (β = 7.896, 95% CI: 5.148-10.644, P<0.001) compared to control. Monotherapy groups showed intermediate effects (dapagliflozin: β = 4.012, P<0.001; linagliptin: β = 3.598, P = 0.002).

**Table 4:** Multiple linear regression analysis of factors associated with change in time in range (ΔTIR).

Independent variable	β Coefficient	Standard Error	Standardized β	t value	P value	95% CI
Constant	8.451	3.102	—	2.724	0.007	(2.325, 14.577)
Treatment group^a^						
Dapagliflozin	4.012	1.118	0.253	3.589	<0.001	(1.802, 6.222)
Linagliptin	3.598	1.165	0.227	3.089	0.002	(1.292, 5.904)
Combination	7.896	1.389	0.443	5.685	<0.001	(5.148, 10.644)
Baseline parameters						
HbA1c, %	1.802	0.488	0.271	3.691	<0.001	(0.839, 2.765)
ALT, U/L	−0.192	0.056	−0.250	−3.429	0.001	(−0.302, −0.082)
UACR, mg/g	−0.023	0.008	−0.188	−2.875	0.005	(−0.039, −0.007)
Serum creatinine, μmol/L	−0.099	0.034	−0.216	−2.912	0.004	(−0.166, −0.032)
Age, years	−0.091	0.041	−0.147	−2.220	0.028	(−0.172, −0.010)

Model statistics: Adjusted R² = 0.428, F = 16.24, P<0.001. All variance inflation factors <3.

^a^Reference category: Control group. ΔTIR calculated as TIR at week 12 minus baseline TIR. Abbreviations: ALT, alanine aminotransferase; CI, confidence interval; HbA1c, glycated hemoglobin A1c; TIR, time in range; UACR, urinary albumin-to-creatinine ratio.

Baseline metabolic status significantly influenced TIR improvement potential. Higher baseline HbA1c predicted greater ΔTIR (β = 1.802, P<0.001), suggesting enhanced treatment responsiveness in poorly controlled patients. Conversely, baseline hepatorenal dysfunction markers negatively predicted ΔTIR, including ALT (β = -0.192, P = 0.001), UACR (β = -0.023, P = 0.005), and serum creatinine (β = -0.099, P = 0.004). Age demonstrated modest negative association with ΔTIR (β = -0.091, P = 0.028), indicating attenuated treatment response in older participants. These findings suggest that patients with preserved organ function achieve superior glycemic stability improvements, emphasizing early intervention importance.

## Discussion

This prospective observational study demonstrated that combination therapy with dapagliflozin and linagliptin provided superior improvements in glycemic control, blood pressure, lipid profiles, and hepatorenal function compared to monotherapy in patients with T2DM and hypertension. The magnitude of TIR improvement with combination therapy—reaching 94.86% compared to approximately 80% with monotherapy—represents a clinically meaningful advancement in glycemic stability.

The combination therapy achieved comprehensive glycemic optimization, improving both average glucose levels and glycemic variability. The observed TIR increase exceeded that seen with either monotherapy, supporting the complementary mechanisms of SGLT-2 and DPP-4 inhibition. Dapagliflozin provides consistent glucose reduction through urinary excretion, while linagliptin modulates postprandial glucose excursions through enhanced incretin signaling (15, 16). This dual mechanism likely explains the superior TIR achievement, consistent with previous findings that combination SGLT-2/DPP-4 therapy enhances glycemic control precision (17).

Blood pressure and lipid improvements were most pronounced with combination therapy. The observed systolic blood pressure reduction to 124 mmHg represents achievement of guideline-recommended targets for diabetic patients. While dapagliflozin’s diuretic and natriuretic effects directly lower blood pressure and may reduce hepatic lipogenesis (18), linagliptin contributes indirectly through improved glycemic control. These findings align with the CARMELINA trial’s demonstration of metabolic syndrome improvement with linagliptin therapy (19).

The combination therapy group demonstrated the most substantial hepatorenal benefits, with ALT and AST reductions exceeding those achieved with monotherapy. The L/S ratio improvement above 1.0 in the combination group suggests reversal of hepatic steatosis, a finding with important clinical implications given the high prevalence of metabolic dysfunction-associated fatty liver disease in T2DM. Previous studies have shown that dapagliflozin reduces hepatic fat accumulation through decreased glucose uptake and lipogenic enzyme inhibition (20), while linagliptin suppresses inflammatory cytokine release (21). The synergistic hepatoprotective effect observed here is consistent with reports of enhanced anti-fibrotic effects with combined SGLT-2/DPP-4 therapy (22).

Renal outcomes similarly favored combination therapy, with greater UACR reduction and eGFR improvement than monotherapy. These findings align with established renoprotective mechanisms: dapagliflozin reduces intraglomerular pressure through tubuloglomerular feedback restoration, as demonstrated in the DAPA-CKD trial showing 36% reduction in renal function deterioration risk (23), while linagliptin ameliorates hyperfiltration and improves renal hemodynamics (24, 25). The combined approach appears to provide additive renoprotection, particularly relevant for this high-risk population.

The correlation analysis revealed important associations between TIR and organ function markers, suggesting that glycemic stability improvements parallel hepatorenal protection. The negative correlations between TIR and liver enzymes, along with positive correlation with L/S ratio, indicate that reduced glycemic variability associates with improved hepatic health. Similarly, the relationships between TIR and renal parameters support glycemic control as a modifiable factor for renal preservation.

The regression analysis identified combination therapy as the strongest predictor of TIR improvement, while baseline organ dysfunction negatively predicted response magnitude. This finding has practical implications: patients with preserved hepatorenal function may achieve optimal benefits from combination therapy, emphasizing early intervention importance. The positive association between baseline HbA1c and TIR improvement suggests that poorly controlled patients have greater potential for improvement, though baseline organ dysfunction may limit this response.

This study’s strengths include comprehensive metabolic assessment, use of CGM-derived TIR as a primary outcome, and evaluation of multiple organ systems. The prospective design with standardized protocols enhances reliability. However, several limitations warrant consideration. The single-center design and 12-week duration may not capture long-term outcomes or generalizability to diverse populations. The observational nature precludes causal inference, though the consistent findings across multiple parameters support the observed associations. The CT-based hepatic steatosis assessment, while practical, lacks the precision of magnetic resonance imaging-proton density fat fraction (MRI-PDFF). Additionally, the absence of detailed safety monitoring, including hypoglycemia incidence and urinary tract infections, limits comprehensive risk-benefit assessment.

The lack of histological evaluation prevents assessment of hepatic inflammation and fibrosis changes. Future studies incorporating liver biopsy or advanced imaging techniques would provide deeper mechanistic insights. The study also did not examine potential biomarkers of treatment response, which could guide personalized therapy selection. Investigation of genetic or metabolic predictors of combination therapy response represents an important future direction.

## Conclusion

Combination therapy with dapagliflozin and linagliptin demonstrated superior efficacy compared to monotherapy for improving glycemic control, blood pressure, lipid profiles, and hepatorenal function in patients with T2DM and hypertension. The achievement of 94.86% TIR with combination therapy, along with significant organ protection benefits, supports this approach for comprehensive metabolic management. The identified predictors of response, particularly the negative impact of baseline organ dysfunction on treatment efficacy, emphasize the importance of early intervention. These findings suggest that combination SGLT-2/DPP-4 inhibitor therapy represents an effective strategy for optimizing outcomes in this high-risk population, though longer-term studies are needed to confirm sustained benefits and safety.

## Data Availability

The raw data supporting the conclusions of this article will be made available by the authors, without undue reservation.
